# Atmospheric inversions for carbon dioxide and methane at a national scale

**DOI:** 10.1093/nsr/nwaf063

**Published:** 2025-02-21

**Authors:** Frédéric Chevallier, Grégoire Broquet

**Affiliations:** Laboratoire des Sciences du Climat et de l'Environnement, LSCE/IPSL, CEA-CNRS-UVSQ, Université Paris-Saclay, France; Laboratoire des Sciences du Climat et de l'Environnement, LSCE/IPSL, CEA-CNRS-UVSQ, Université Paris-Saclay, France

## Abstract

This paper outlines the current state of national-scale atmospheric inversion, at a time when not only the volume of observational data, but also institutional expectations, are increasing considerably.

For decades, the inversion of carbon dioxide (CO_2_) atmospheric transport global models has been mainly used to analyse atmospheric measurements for the identification of the latitudinal distribution of natural emissions and absorptions. The steady growth of the CO_2_ observation system and the launch of dedicated satellite missions from the end of the 2000s have allowed characterization of the longitudinal distributions as well and have stimulated the development of regional limited-area atmospheric inversion systems. The geographical concentration of fossil emissions motivated the establishment of atmospheric inversion for urban or industrial plumes specifically. Similar studies for methane (CH_4_) started later along the same path. They were both slowed down by the later development of the surface network and by uncertainty in methane oxidation, and facilitated by strong local CH_4_ anthropogenic emissions that were hardly documented on the basis of traditional emission inventories but clearly visible in images of the methane column.

Since 2006, already, the United Nations Framework Convention on Climate Change (UNFCCC) has encouraged the use of atmospheric inversion in the evaluation of national emission inventories for greenhouse gases (GHGs). This recommendation has been followed in some countries such as China [1], at least in research mode and mostly for CH_4_, but also for the CO_2_ fluxes associated with land use, land use change and forestry. The analysis framework that was designed by the corresponding studies provides the foundation for a wider institutional adoption of the recommendation. The World Meteorological Organization is working to coordinate inversion efforts to assess the impact and overall effectiveness of mitigation endeavors that are undertaken by the parties to the UNFCCC's Paris Climate Agreement.

By contributing to the evaluation of emission inventories, atmospheric inversion can provide reference values that guide the preparation of inventories and the improvement of the underlying models. Atmospheric inversion can also integrate the inventory models directly in its estimation process and therefore directly inform about the mechanisms and parameters that are controlling the GHG emission and absorption fluxes that are often crudely represented or defined. These insights can in turn guide further improvement of bottom-up estimates for key sectors, such as methane emissions in China from coal mining and rice paddies or terrestrial CO_2_ sinks.

While the core methodology for compiling detailed reference inventories for the UNFCCC and its Global StockTake is expected to remain stable in the coming decade, atmospheric data gradually enhance and complement these inventories.

In this respect, atmospheric inversion is entering a golden age, with space agencies and even private companies that are vying to monitor GHG columns around the world with ever greater spatial resolution and density. Reducing the ground footprint of observations makes it easier to attribute atmospheric signals to territories or processes, but also facilitates ‘hunting the holes’ between the clouds that interfere with the GHG signal in the spectral measurements. The observation of methane already benefits, for instance, from the contribution of the European Copernicus Sentinel-5P satellite, with a 2600-km-wide swath, and from several private satellites with sub-km resolution, including Xiguang-1 04, China's first high-resolution methane-monitoring commercial satellite that was launched in November 2024. China pioneered active remote sensing for CO_2_ with a lidar on board the AESM satellite that was launched in April 2022, while a partnership between France and the UK will test the benefit of radiance observations in a new spectral band to mitigate aerosol interference in the retrieval of the CO_2_ column with the launch of the MicroCarb satellite in mid-2025. The European Copernicus Anthropogenic Carbon Dioxide Monitoring (CO2M) program will be made up of a constellation of imaging satellites for CO_2_ and CH_4_ that will be deployed from 2027. CO2M will yield an unprecedented number of CO_2_ column retrievals in particular, thanks to 240-km-wide swaths. The data count of the planned missions obviously remains theoretical, as few spectrometers and processing algorithms are able to meet the stringent requirements of inverse modeling in practice. Even today, the most accurate satellite CO_2_ retrievals from passive instruments likely suffer from large residual biases in the presence of aerosols.

The growing diversity and performance of computer processing units (central, graphics, tensor, quantum, etc.) offer unprecedented power to handle this growing wealth of observation data, while new computer-programming frameworks and the development of artificial intelligence pave the way for versatile or hybrid workflows that can merge them with other types of information.

Regional systems that are developed and run with local expertise are the most obvious type of inverse modeling systems for national-scale estimates of GHG emissions and absorptions. Geographic information can allow some distinction between natural and anthropogenic surface fluxes in their results, and even some sectoral attribution, as illustrated by recent studies for China [2]. The co-assimilation of related tracers such as isotopes or some pollutants can also help. In Europe, systems that target the national GHG emissions are now run at resolutions of 50–10 km, which allows, in principle, the analysis of both large-scale variations in the concentrations across the countries and specific plumes from the largest anthropogenic emission hotspots.

However, the annual budgets of continental- to country-scale GHG fluxes from the regional inversions still do not constrain their boundary conditions well enough and still suffer from atmospheric transport modeling errors. They also need to reach a km-scale resolution to separate highly localized CO_2_ anthropogenic emissions and regional-scale flux signals.

The challenge that is posed by the lateral boundary conditions of the regional systems reinforces the role of global systems that conserve mass at all scales. The one-degree global inversion resolution that is used within the European operational Copernicus service is not far from the resolution of some regional systems and therefore offers the near prospect of direct comparisons with aggregated ground-truth flux measurements, which is likely to represent a critical step in the uptake of global inverse modeling beyond the scientific community.

The challenge that is posed by the emission hotspots also motivates local-scale approaches in which observations of plumes from individual hotspots are processed with km-scale 3D or 2D transport modeling capabilities, sometimes now re-expressed with artificial intelligence. They may rely on the gradual increase in the spatial resolution of the regional systems or on advanced couplings between regional and local-scale inversion systems. However, the spatial and temporal representativeness of the GHG plumes that is observed by current and planned spatial imagery remains limited and very few cities are currently equipped with urban networks. The full coverage of the anthropogenic emissions in a country has thus to rely on complementary information: observations of related tracers, spatial inventories and activity data with high spatial and temporal resolution, etc.

The uncertainty in atmospheric transport modeling is strongly connected to the quality of wind information, highlighting a need to couple atmospheric inversion systems and numerical weather prediction ones. Such a perspective is highlighted by the leadership taken by the European Centre for Medium-Range Weather Forecasts in the development of an anthropogenic GHG emissions monitoring and verification support capacity.

Addressing these various challenges will be particularly important for countries with a very heterogeneous carbon budget like China, which has both a wide range of ecosystems with rapid greening trends and intense anthropogenic emissions, not only from fossil fuel. International standards to objectively quantify how well they have been addressed based on internal consistency diagnostics and independent and open-access reference data will be as important. Indeed, the diversity of inverse modeling estimates can generate confusion rather than transparency, as was illustrated by a recent controversy over the size of China's terrestrial carbon sink [3].

Fortunately, some of the challenges will be reduced by future satellite missions. The increasing amount of high-quality satellite data will reduce the computational cost of global and regional inversions by allowing data assimilation windows to be considerably shortened and reducing the role of their prior uncertainty covariance matrix whose complexity can be challenging with increasing dimension. It will also reduce the impact of large-scale transport model errors, bring some information on local winds directly and inform about GHG emissions and absorptions over a wide range of scales at once.
